# Dedicated Endoscopy for Barrett's Oesophagus With Higher Dysplasia Yield May Reduce Seattle Protocol Biopsies: Results From UK Multicentre Study

**DOI:** 10.1002/ueg2.70270

**Published:** 2026-07-29

**Authors:** Champika Gamakaranage, James Britton, Elizabeth Ratcliffe, Neeraj Prasad, Richard Keld, Mark Murgatroyd, Shanmugasundaram Balakrishnan, Nazar Aslam, Vinay Sehgal, Jenni Marsh, Daniel Plant, Matthew Johnston, Stefanos Kateroglou, Jake Baker, Hannah Morgan, Claudia Cipriano, Anjan Dhar, Shaheen Hamdy, John McLaughlin, Yeng Ang, DEBO group

**Affiliations:** ^1^ Division of Diabetes, Endocrinology and Gastroenterology School of Medical Sciences, Faculty of Biology Medicine and Health The University of Manchester Manchester UK; ^2^ Wrightington Wigan and Leigh Teaching Hospitals NHS Foundation Trust` Wigan UK; ^3^ Northern Care Alliance NHS Foundation Trust Salford UK; ^4^ Bolton NHS Foundation Trust Farnworth UK; ^5^ University College London Hospitals NHS Foundation Trust London UK; ^6^ University College London London UK; ^7^ County Durham and Darlington NHS Foundation Trust Darlington UK; ^8^ Teesside University Middlesbrough UK

**Keywords:** Barrett's oesophagus, dedicated service, dysplasia detection, endoscopic surveillance, key performance indicators

## Abstract

**Background:**

Dedicated service for Barrett's oesophagus (BO) surveillance may be more effective than conventional service, according to some single centre studies.

**Objective:**

To determine whether the surveillance of BO through a dedicated service can improve key performance indicators (KPIs), and dysplasia detection rate (DDR) compared with conventional service in a multi centre study.

**Methods:**

A retrospective cohort study was conducted across 6 NHS‐hospitals, capturing BO surveillance data over 8 years. Factors associated with DDR were assessed by logistic regression.

**Results:**

There were 1037 dedicated and 976 conventional surveillance procedures (*N* = 2013), male: female ratio = 2.2:1; mean age = 64.4 (SD ± 12.2) years; mean maximum length of BO = 4.1 cm (range: 0–18 cm). All the KPIs and DDR were significantly higher in the dedicated service (DDR = 6.9%) than in the conventional service (DDR = 2.8%), *p* < 0.001. The use of narrow band imaging (NBI) and acetic acid chromoendoscopy (AAC) and sedation were high, and the complication rate was significantly lower in the dedicated service. The lesion recognition was significantly associated with DDR (OR = 6.9). Surprisingly, Seattle biopsy protocol adherence showed no correlation with DDR (OR = 0.47).

**Conclusion:**

The dedicated service provides higher quality endoscopy, and a higher yield of early neoplasia. It uses more sedation, advanced imaging, and detects more lesions than conventional services. Thus, the dedicated surveillance could potentially replace time and cost consuming Seattle biopsies with targeted biopsies of visible lesions. In future, this may become easier with the use of artificial intelligence for lesion detection (CADe).

## Key Summary

- Summarise the established knowledge on this subject
  ◦ Current evidence supporting dedicated service for BO surveillance comes from few small‐medium single centre cohort studies and a pilot RCT.
  ◦ These early studies suggest that a dedicated service improves key performance indicators (KPI) and dysplasia detection than a non‐dedicated service.
- What are the significant and/or new findings of this study?
  ◦ This is the largest multicentre comparison of two services spanning across tertiary referral centres (with enriched population having a higher dysplasia prevalence) and medium to small hospitals (with non‐enriched population), improving generalisability.
  ◦ Lesion recognition emerges as a key determinant of DDR, whereas Seattle protocol adherence doesn't, highlighting the importance of skill development in endoscopists over blind biopsy protocol adherence.
  ◦ This is the first study to effectively compare complication rates between two services.

## 1 Introduction

Oesophageal adenocarcinoma (OAC) is growing fast in Europe with a poor 5‐year survival when it is invasive. Barrett's oesophagus (BO), with an overall prevalence of 1%–2%, could progress to OAC through stages of dysplasia: low grade (LGD) or high grade (HGD) [1, 2, 3]. Endoscopy remains the investigation of choice for surveillance, although more, less invasive methods such as capsule sponges are emerging [4, 5, 6, 7].

Endoscopic assessment needs a specific set of skills to ensure high‐quality and yield of early neoplastic lesions. The quality of endoscopic surveillance is assessed with key performance indicators (KPI) as described in the British Society of Gastroenterology (BSG) guidelines, and in the dedicated endoscopy BO (DEBO) pilot randomised controlled trial (RCT) [2, 8]. However, this guideline or the current European Society of Gastrointestinal Endoscopy (ESGE) guidelines do not specify a dedicated surveillance service for BO. Both guidelines assume that the skills of an endoscopist are optimal for the detection of early neoplasia; furthermore, they emphasise quality assurance and appropriate surveillance intervals but leave the service configuration and training pathways open to local variability [2, 3]. This paper explores strategies to enhance the outcome of BO surveillance through dedicated service by comparative analysis between dedicated service and conventional (non‐dedicated) service.

BO surveillance practices have improved since their recommendation a decade ago with the advancement in endoscopy equipment, special imaging techniques and the development of skills and experience in endoscopists. However, most available data comes from the single expert centre studies or from data before these practices were well established. These include few single centre cohort studies and one pilot RCT which have demonstrated benefits of dedicated service over conventional service by having better quality of endoscopy, better adherence to guidelines and higher dysplasia detection rates (DDR) [8, 9, 10, 11, 12].

The aim of this study was to assess whether dedicated service for BO surveillance is more effective in quality and yield (measured by KPI and DDR) compared to conventional service in multiple surveillance centres in the UK, over 8‐years, where both services have existed.

### 1.1 Objectives

#### 1.1.1 Primary Objective

To assess whether dedicated endoscopic surveillance of Barrett's oesophagus has better quality and yield compared with conventional non‐dedicated surveillance.

#### 1.1.2 Secondary Objectives

1. To compare the quality of endoscopy of the two services using KPIs.
2. To compare the use of advanced imaging techniques (narrow band imaging (NBI) and Acetic Acid Chromoendoscopy (AAC)) in each of these two services.
3. To compare the neoplasia yield of two services by means of DDR.
4. To compare the rate of occurrence of complications in the two services.
5. To identify and assess the strength of associations of KPIs associated with dysplasia detection.

## 2 Materials and Methods

### 2.1 Study Design

A multicentre retrospective cohort study on BO surveillance comparing dedicated service versus conventional service.

### 2.2 Study Setting

The study was conducted across 6 hospitals, representing a mixture of tertiary referral centres (3), and medium/small hospitals (3) in the UK between January 2017 and 2025, in which the surveillance practices were technically similar and followed the BSG‐2014 guidelines.

The dedicated service was defined as endoscopic BO surveillance performed by an endoscopist with enhanced training for neoplasia detection and/or supervised by a gastroenterologist with experience in Barrett's endotherapy and performing regular surveillance endoscopy (> 100/year). In conventional service, surveillance is conducted within a mixed endoscopy list by an endoscopist without such specific BO‐focused training and having no formal training on lesion recognition [8].

### 2.3 Participants and Procedures

Real world NHS electronic patient records of BO surveillance endoscopy conducted within the period of study were accessed and selected using eligibility criteria.

#### 2.3.1 Inclusion Criteria

1. Consecutive procedures on patients with suspected (endoscopic BO without histological confirmation of IM) or confirmed (histologically IM) BO undergoing routine planned endoscopic surveillance per BSG guidelines.
2. Endoscopic assessment of BO with surveillance intent while conducting the endoscopy procedure for other indications.

#### 2.3.2 Exclusion Criteria

1. Those already diagnosed with BO with HGD/OAC or previously treated by surgery or endotherapy.
2. Surveillance procedures conducted before first January 2017.

### 2.4 Variables

Demographic data (age and sex), clinical data (Barrett's length) and surveillance data (KPIs, DDR and other) were collected. Surveillance data were extracted from endoscopy and histology reports and are enumerated Table 1. Patients' clinic notes and referral letters were accessed where clarifications were needed.

**TABLE 1:** Summary of participant characteristics, key performance indicators (KPI), and other important parameters.

Parameter				
Participant characteristics		DS	NS	Total
Age		64.7 (± 12.0)	64.0 (± 12.5)	64.4 (± 12/2)
Sex	Male	724 (69.8%)	655 (67.1%)	1379 (68.5%)
	Female	313 (30.2%)	321 (32.9%)	634 (68.5%)
Maximum length of BO		4.2 (± 3.2)	3.9 (± 2.9)	4.06 (± 3.2)
**NHS hospital**		**DS procedures [patients]**	**NS procedures [patients]**	**Total procedures [patients]**
Wrightington Wigan and Leigh (WWL)		505 [468]	344 [319]	849 [787]
Salford Royal Hospital (SRFT)		265 [208]	292 [229]	557 [437]
Burry Rochdale and Oldham (BRO)		115 [97]	197 [84]	312 [181]
County Durham and Darlington (CDD)		94 [89]	44 [44]	138 [133]
Bolton NHS FT		45 [40]	92 [64]	137 [104]
University College London (UCL)		13 [13]	7 [7]	20 [20]
*Subtotal (%)*		*1037 (51.5%)*	*976 (48.5%)*	**2013 [1662]**
**KPI**		**DS (%)**	**NS (%)**	***p***
Hiatus hernia delineation		746 (72.1)	289 (27.9)	< 0.001
Prague classification		1004 (96.8)	868 (89.0)	< 0.001
Island description		200 (19.3)	53 (5.4)	< 0.001
Lesions description		227 (21.9)	61 (6.3)	< 0.001
Paris classification		63 (6.4)	5 (0.5)	< 0.001
Seattle protocol adherence		959 (92.6)	747 (76.6)	< 0.001
Targeted biopsies		249 (24.0)	787 (76.0)	< 0.001
Barrett's inspection time (1 min/cm)		395 (38.1)	7 (0.7)	< 0.001
Other parameters				
Use of sedation		522 (51.2%)	501 (48.9)	0.019
NBI use		938 (90.7)	325 (33.3)	< 0.001
AAC use		674 (65.1)	44 (4.5)	< 0.001
DDR with IDD		127 (12.5)	76 (8.2)	0.0025
DDR without IDD		70 (6.9)	26 (2.8)	< 0.001
Complications		3 (0.3)	11 (1.1)	0.046

*Note:* Bold and italic indicates the subtotal and total values.

Abbreviations: AAC = acetic acid chromoendoscopy, BO = Barrett's oesophagus, DDR = dysplasia detection rate, DS = Dedicated service, IDD = indefinite for dysplasia, NBI = Narrow band imaging, NS = Non‐dedicated service.

### 2.5 Outcome Measures

Primary: Assessment of quality of Barrett's surveillance endoscopy by KPIs and yield by DDR.

#### 2.5.1 Secondary

- Rates of use of advanced imaging (NBI/AAC).
- Documentation of description(s) of visible lesions.
- Rates of detection of IM, dysplasia (LGD/HGD) and OAC at histology.
- Rate of sedation use and occurrence of complications.

### 2.6 Endoscopic Assessment

Endoscopic assessment and biopsy procedures followed the BSG guidelines, and our practice is summarised below.

**FIGURE 1:**
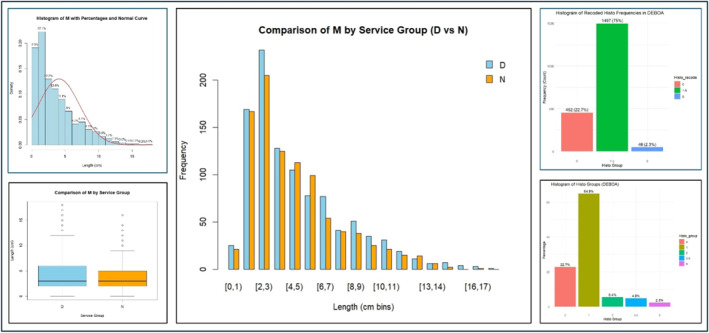
Graphical description of Maximum length (M) of BO and histology in DEBO dataset. Upper left—Histogram shows the distribution of maximum length of BO (M) per centimetre in the total procedures. Lower left—Comparison of means of M in the two services (D = dedicated, N = nondedicated). There is a marginal statistical difference between the two means (*p* = 0.05). Middle—Distribution of M of BO in two services showing comparable distribution. Upper right—Distribution of histology showing a positive test in 75% of procedures. Lower right—Distribution of histology when intestinal metaplasia (1), indefinite for dysplasia (2), low grade dysplasia + high grade dysplasia + oesophageal adenocarcinoma (3–5), and no biopsies taken (9) are plotted. DEBOA = DEBO dataset, “Histo” = histology.

**FIGURE 2:**
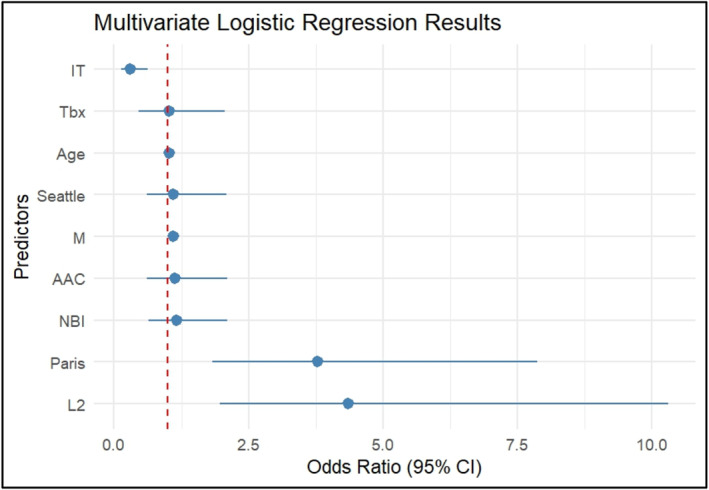
Forest plot visualising the odds ratios (ORs) and 95% confidence intervals (CIs) for predictors in the multivariate logistic regression model. The vertical red dashed line at OR = 1 represents the null effect—values to the right: increased odds, while values to the left: decreased odds. AAC = acetic acid chromoendoscopy, IT = inspection time, L2 = lesions documentation, M = maximum length, NBI = narrow band imaging, Paris = Paris classification documentation, Tbx = target biopsy.

**FIGURE 3:**
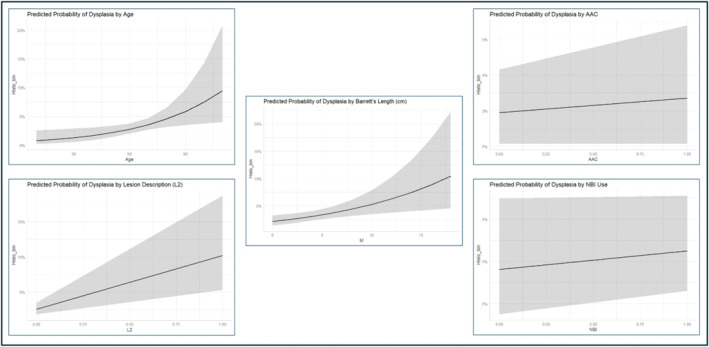
Predictive probability of dysplasia detection against age, length of BO, lesion description, AAC and NBI. AAC = acetic acid chromoendoscopy, BO = Barrett's oesophagus, NBI = narrow band imaging.

**FIGURE 4:**
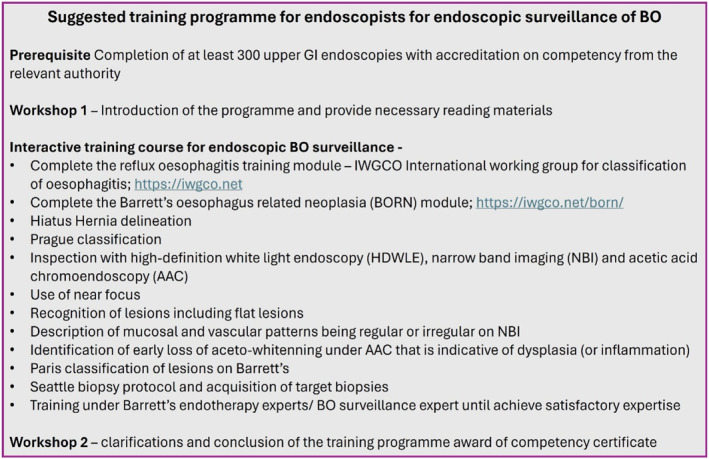
The training programme for Barrett's surveillance.

*Equipment:* Olympus 290 series, Olympus GIS XZ 1200 zoom scope and Olympus 1500 series endoscopes were used in the 6 centres. *Endoscopists:* All the endoscopists were accredited endoscopists in the UK consisting of consultant gastroenterologists, trainee gastroenterologists, clinical fellows and nurse endoscopists. *Patient preparations* were according to the routine endoscopy recommendations, and the use of local anaesthesia or sedation was according to the clinical indications and choice of the endoscopists and patients. *Mucosal preparation:* Water, simethicone and N‐acetylcysteine were commonly used to reduce mucus and bubble formation. *Measurements* were taken from important landmarks and expressed in centimetres from incisors. The delineation of the gastroesophageal junction (GOJ) and diaphragmatic pinch (DP) were noted. The distance between the DP and the top of the gastric folds (denotes GOJ) was expressed as the length of the hiatus hernia (HH). *Inspection of Barrett's mucosa:* High‐definition white light endoscopy (HDWLE) was used in all cases. Barrett's mucosa was first inspected with HDWLE in an overview assessment and measurements were taken. Any macroscopically obvious lesions were noted and further characterised by near‐focus view (magnification endoscopy). *Use of NBI and AAC:* NBI was used to assess any mucosal pit and vascular patterns in the BO mucosa. Irregular patterns may indicate dysplasia and warrant target biopsies. Spray of 1.5%–3% acetic acid (AA) to the Barrett's mucosa was performed using a spray catheter followed by washing of extra dye with a gentle water jet. Columnar epithelium, in contrast to native stratified squamous epithelium, accentuates with AA and immediately stands out from metaplastic Barrett's. Dysplastic (or inflamed) areas absorb AA early (in 2 minutes or early) and the areas of early loss of aceto‐whitening (ELOW) were biopsied. *Lesion recognition and Paris classification:* Macroscopic lesions appear as polyps, nodules, or mucosal irregularities or nodularity, and they were described using Paris classification. Macroscopically inapparent, flat lesions were characterised by near focus using HDWLE/NBI/AAC. The position of the lesions was described with distance from the incisors and clock‐face position. See Supporting Information S1: Figures 1, 2, 3 and 4.

### 2.7 Biopsy Protocol

Target biopsies: lesions were biopsied and sent in separate cassettes, keeping in mind that the localisation is used for future surveillance or endotherapy on target areas.

Random biopsies: structured random biopsies by the Seattle biopsy protocol were followed in all centres, in which the 4 quadrant biopsies (4QB) were taken every 2 cm intervals of the Barrett's mucosa starting 1 cm proximal to the GOJ.

### 2.8 Histology

The modified Vienna classification was used to report the histological findings [13]. This reports whether there were columnar epithelium, intestinal metaplasia (IM), and dysplasia (indefinite for dysplasia (IDD), LGD, and HGD) or OAC) [2, 3, 14]. We used the highest reported stage of dysplasia among all the biopsies as the final diagnosis for the report. The results were independently interpreted by two expert upper gastrointestinal histopathologists. When dysplasia was present, p53 marker assessment was frequently performed, enhancing the accuracy of the final diagnosis, although this data was not collected in the current study.

### 2.9 Ethics Approval

This study was first registered in the audits and research and innovation departments of Northern Care Alliance NHS Foundation Trust (audit number: 2024‐214) and health improvement project (HIP) number: 24HIP71. Subsequently, other centres were recruited with each centre individually registered as a local audit. Therefore, research ethics committee approval was not required.

### 2.10 Patient Consent

The consent was not required as the data were collected retrospectively and anonymously and conducted as a multicentre audit.

### 2.11 Data Collection

Research team members who collected data were experienced endoscopists or doctors with an interest in BO to ensure quality and participated in a training session with CG (online/face to face) to ensure uniformity, minimising bias. *Identification and data entry:* eligible data were entered into standardised spreadsheets (Microsoft Excel, Microsoft Corp Inc.) and anonymised by removing patient identification information such as names and hospital/NHS numbers. *Data transfer and storage:* anonymised data sheets were encrypted with passwords and transferred to the primary centre using secure NHS emails. The data is processed, and will be securely stored for 5 years, following the data management plan (DMP) of the University of Manchester.

### 2.12 Statistical Analysis

Analysis was conducted using RStudio version:2025.09.0 + 387. The characteristics of the patients were described demographically, and a per‐procedure analysis was conducted. KPIs and other indicators of the two arms were compared with independent samples *t*‐test for continuous variables and the Chi‐Square test or Fisher's exact test for categorical variables. A *p* value of < 0.05 was considered statistically significant. Missing data was < 3% and seems random and ignored during analysis. The univariate and multivariate logistic regression analyses were performed to identify the factors associated with DDR.

#### 2.12.1 Sample Size Calculation

This was calculated considering DDR in previous studies [8]. When the DDR in dedicated service is 6.3% and conventional service is 2.7%, a sample size of 691 is needed for each arm to achieve a power of 90% at 5% significance. When 10% attrition is used for potential missing data, approximately 768 procedures are needed per service (*N* = 1536). However, considering the importance of subgroup analysis and generalisability with a multicentre study, we aimed for 2000 total data collection to ensure a statistical rigour.

## 3 Results

Data from 2013 procedures (dedicated service:1037, conventional service:976) performed on 1662 patients were included in this study. Male: female ratio = 2.2:1, mean age = 64.4 (SD ± 12.2) years. The mean maximum length of BO = 4.1 cm (SD ± 3.1): ultrashort (< 1 cm):46 (2.3%), short (1– < 3 cm):773 (39.2%), long (3–9 cm):1009 (51.2%) and ultralong (≥ 10 cm): 91 (4.6%). See Table 1 and Figure 1.

### 3.1 Quality of Endoscopic Surveillance

All the KPIs were significantly higher in the dedicated service group. All endoscopic assessments were done with HDWLE in both dedicated and conventional services and across all the centres. The use of NBI/AAC was significantly higher in the dedicated service than in the conventional service. These findings are summarised in Table 1 and Figure 1.

### 3.2 Yield of Endoscopic Surveillance

#### 3.2.1 Histological Findings

The diagnosis of the BO needed a demonstration of IM in the BO biopsies. When the detection of IM, dysplasia and neoplasia were considered as a positive test, there were 1497 (75%) positive tests across all the surveillances performed and no significant difference between two services observed for the yield of a positive test (dedicated service:77.7% (792/1019), conventional service:75.8% (705/930), *p* = 0.34).

#### 3.2.2 Dysplasia Detection Rate (DDR)

When the DDR was calculated with IDD (i.e., IDD + LGD + HGD + OAC), there was a significantly higher detection rate in dedicated service (127/1019 (12.5%)) than in conventional service (76/930 (8.2%)), *p* = 0.0025. The effect persisted when the DDR is calculation excluding IDD: dedicated service 70/1019 (6.9%), conventional service 26/930 (2.8%), *p* < 0.001. No dysplasia (LGD, HGD or OAC) was detected in those with ultrashort segment BO (< 1 cm). The lesion detection rate (LDR) is another important outcome of endoscopic surveillance although this particular calculation was not accurately achievable in this study due to the lack of information in the endoscopy reports, the lesions documentation may closely resemble LDR.

### 3.3 Factors Associated With Dysplasia Detection

Please see Table 2 and Figure 2. The univariate logistic regression showed that age, maximum BO length, use of NBI/AAC, lesion recognition, Paris classification, and acquisition of target biopsies were associated with increased DDR. However, sex, HH delineation, Prague classification documentation, island description and sedation use were not shown to have a significant association. In contrast, Seattle protocol adherence and inspection time documentation were associated with reduced odds of dysplasia detection. Multivariate logistic regression showed strong associations for dysplasia detection with lesion description and Paris classification. The patients undergoing dedicated service were 3.2 times more likely to have lesion description documented than in conventional service (risk ratio (RR) = 3.22 (95% CI 2.84‐3.66)), with odds of lesion documentation in dedicated service is 8 times higher than conventional service (Odds ratio (OR) = 8.08 (95% CI 6.60–9.88)) and attributable risk (AR) in dedicated service is 47.3% (i.e., If conventional service improved to dedicated service, about 47% more patients would have lesions documented).

**TABLE 2:** Summary of univariate logistic regression.

Predictor	OR (estimate)	95% CI (Lower–Upper)	*p*‐value	Significance
Age (continuous)	1.03	1.01–1.05	0.0018	**Significant**
Sex (male vs. female)	1.37	0.87–2.24	0.191	Not significant
HH delineation	1.30	0.93–1.80	0.124	Not significant
PC documentation	1.05	0.49–2.73	0.913	Not significant
Maximum length (M)	1.15	1.09–1.22	< 0.001	**Significant**
NBI use	1.74	1.10–2.85	0.021	**Significant**
AAC use	1.76	1.16–2.66	0.0073	**Significant**
Island description	0.79	0.42–1.34	0.420	Not significant
Lesion recognition	6.92	4.53–10.6	< 0.001	**Highly significant**
Paris classification	12.2	6.88–21.3	< 0.001	**Highly significant**
Seattle protocol	0.47	0.29–0.79	0.003	**Significant (negatively)**
Target biopsy (Tbx)	2.51	1.80–3.44	< 0.001	**Highly significant**
Inspection time (IT)	0.49	0.25–0.89	0.029	**Significant (negatively)**
Sedation use	1.09	0.84–1.31	0.437	Not significant

*Note:* Bold indicates the items with significance.

Abbreviations: AAC = acetic acid chromoendoscopy, HH = hiatus hernia, NBI = narrow band imaging, PC = Prague classification.

### 3.4 Prediction of Dysplasia Detection Through Associated Factors

Based on the findings of logistic regression, the predictive probability of dysplasia detection can be assessed and is graphically demonstrated in Figure 3.

### 3.5 Number Needed to Scope (NNS)

The number need to test (NNT) or the NNS was 24.4 comparing two services.

### 3.6 Complications

The overall incidence of complications is 0.7% with a significantly lower rate in dedicated service (3/1037 (0.3%)) than in conventional service (11/975 (1.1%)), *p* = 0.046; making dedicated service 3.7 times safer than conventional service. Excess bleeding is the most common complication (9/14), followed by intolerance (4/14) and bradycardia (1/14).

### 3.7 Use of Sedation and Inspection Time

Sedation usage was significantly higher in the dedicated service of 51% than in the conventional service of 46% (*p* = 0.04)). The inspection time documentation was low but much higher in the dedicated service (38%) than in the conventional service (0.7%), *p* < 0.001.

### 3.8 Endoscopists

There were 182 (dedicated service −15/conventional service‐167) endoscopists involved in procedures recorded across 6 centres. The endoscopists are consultant gastroenterologists, trainee gastroenterologists, clinical fellows, nurse endoscopists, surgical endoscopists, and locum endoscopists representing a diverse clinical community.

### 3.9 BO Surveillance Training Programme and Prospective Follow‐Up Study

Following the completion of this retrospective comparative study as a part of the HIP, we initiated a structured training programme for volunteering endoscopists (*n* = 20) in conventional service to improve their BO surveillance skills. The suggested training programme is illustrated in Figure 4. Following completion of the training, we plan for a prospective comparison of pre‐ and post‐training KPIs and DDR to assess whether the training programme is effective.

## 4 Discussion

This large multicentre study showed that dedicated service can deliver a high‐quality endoscopic surveillance for BO, resulting in a higher DDR, which is about 2.5 times higher than that of conventional service. The most important factor associated with DDR was lesion recognition, thereby highlighting the importance of improving the optical skills of endoscopists through training on lesion recognition. The KPIs and other parameters pertaining to the quality of surveillance and DDR have been assessed in previous single centre cohort studies and a pilot RCT has shown results concordant with the current study [8, 9, 10, 11].

The overall effectiveness of dedicated service over conventional service through a higher detection of early neoplasia in BO could lead to a survival benefit of OAC. This has been shown in a large cohort study (*n* = 969 BO patients) from Cambridge conducted over 24 years (1997–2022, median follow‐up of 5.8 years). There was a higher detection rate of HGD 61/969 (6.3%) and OAC 48/969 (4.5%) and low disease specific mortality 3/969 (0.3%) in the hands of this expert centre [15]. However, Barrett's Oesophagus Surveillance Study (BOSS, RCT) did not show a survival benefit in the 2‐yearly surveillance group compared with the Endoscopy at Need arm over a 20‐year follow‐up started since 2009 [16]. The limited quality of endoscopy at this era and the lack of risk stratification may have partially contributed to these results [17]. Therefore, risk stratification and surveillance of high‐risk patients through a dedicated service may have the potential to demonstrate a survival benefit in an adequately large sample. Our study indicates that the increase in the number of neoplastic lesions in the dedicated service would result in endoscopic treatment of these patients, reducing their risk of progression to advanced OAC and thereby potential to influence survival.

The quality of surveillance endoscopy in BO is a collective result of all KPIs, similar to that of colonoscopy where the adenoma detection rate is dependent on the additive effect of several measures (withdrawal time, use of antispasmodic, patient position change and rectal retroflexion), as described in the quality improvement in colonoscopy (QIC) study [18]. We suggest adherence to the KPIs as a package to improve the quality of BO surveillance.

The use of NBI and AAC was recommended by the American Society of Gastroenterology (ASGE) as the advanced techniques to use in BO surveillance as they fulfil the prevention and incorporation of valuable endoscopic innovations (PIVI) threshold for detecting HGD/early cancer [19]. NBI and AAC were used more in dedicated service and have been shown to improve DDR, highlighting their benefits. The use of near focus imaging is important in lesion recognition, especially those with muco‐vascular irregularities and flat lesions. However, application of near focus was not mentioned in most reports. Therefore, these data was not collected in the current study. Future prospective studies should assess this with structured data collection materials.

The DDR in general is around 3%–5% in BO surveillance [12], which is consistent with our overall DDR (LGD + HGD + OAC) of 4.8% although it is significantly higher in dedicated service (6.9%) than in conventional service (2.8%) (*p* < 0.001). The increase in DDR by 4.1% in dedicated service may be due to the combined effect of higher KPIs, longer Barrett's inspection time, higher use of sedation, and lesion recognition with acquisition of more target biopsies. Regression analysis showed that lesion recognition is the most important independent determinant of DDR. The Seattle protocol adherence was associated with significantly lower DDR, indicating that blind random biopsies may be ineffective in detecting early neoplasia in BO, particularly in the conventional service group. Given the compelling evidence favouring dedicated service, it would be prudent to reassess those who underwent surveillance in conventional service, through dedicated service in their next surveillance.

However, the relationship between lesion recognition, Paris classification, target biopsies and Seattle adherence requires careful interpretation. Since apparent lesions such as nodules and polyps could be difficult to miss in either service, a dedicated service may have detected more flat lesions enhanced by advanced imaging, magnification endoscopy and higher inspection time, although such details were not adequately available in endoscopy reports. Conversely, a negative relationship was seen with Seattle protocol adherence to DDR. One explanation is deliberate avoidance of Seattle biopsies when lesions are already detected, especially by experts in the dedicated arm. This could indicate the importance of lesion detection and target biopsies, which could reduce the time and cost of surveillance compared with Seattle biopsies, especially if artificial intelligence (AI) could be incorporated, directing BO surveillance to a cost‐effective path. A prospective study to collect this information is recommended for definite conclusions, particularly with standardised detailed reporting of procedures. The data analysis intentionally followed a per‐procedure analysis rather than per‐person analysis, taking into consideration that each surveillance represents fresh surveillance with its own independent KPIs. Thus, a per‐person analysis and patient wise sensitivity analyses were not feasible. Endoscopy is a safe procedure with a low complication rate; bleeding 0.1% and perforation 0.03% [20, 21]. The overall complication rate in the current study was 0.7%, including those events leading to abandonment of the procedure. As the number of complications is small, no further meaningful comparisons could be made.

To the best of our knowledge, this is the largest multicentre study comparing dedicated service versus conventional service for BO surveillance to date, but our results are limited by its retrospective nature of the study. Furthermore, the imbalance in the number of endoscopists in the two services could introduce confounding results, although this is expected in this real‐world retrospective study. An adequately powered multicentre RCT is needed to validate these findings and the true benefits of survival, cost effectiveness and patient reported outcomes related to dedicated service. Our study did not assess the post endoscopy upper gastrointestinal cancer rate (PEUGIC) in the two services, and this also needs to be assessed as an important parameter to compare the two services in future. It is worth highlighting the importance of mucosal preparation, which was lacking in our study, as it plays a pivotal role in improving lesion recognition.

## 5 Conclusion

Endoscopic surveillance of BO through dedicated service provides a high‐quality endoscopy, lower complications and higher yield. Lesion recognition is an important parameter that is associated with DDR. Prospective studies to assess whether targeted biopsies could safely reduce Seattle adherence and reduce cost and resource exhaustion are recommended as future directions. We suggest training endoscopists to improve KPIs, use of advanced imaging techniques, recognition of lesions and acquisition of more target biopsies. Further studies are needed to demonstrate the survival benefit and cost‐effectiveness of modern dedicated service in BO surveillance with recruitment of stratified high‐risk patients in contrast to “blanket” surveillance. Incorporation of KPIs and other important parameters in an AI computer aided detection (CADe) and characterisation (CADx) model may provide an additional opportunity to improve DDR in the future.

## Funding

The authors have nothing to report.

## Conflicts of Interest

Y.A. and E.R. received funding from Medtronic and Y.A. has research funding from the Cancer Research UK. S.H. is the director and chief scientific officer for Phagenesis Ltd, a company that focusses on dysphagia treatment and holds founder shares and he holds stocks and shares in Anisys Ltd, a company that focusses on anorectal diagnostics and biofeedback. Other authors declare no competing interests.

## Permission to Reproduce Material From Other Sources

The authors have nothing to report.

## Supporting information


Supporting Information S1

## Data Availability

The data will be kept secured in the primary centre for 5 years before they are destroyed. The raw data underlying this study are available from the corresponding author upon reasonable request.
