# Supplementary material for: Dedicated Endoscopy for Barrett's Oesophagus With Higher Dysplasia Yield May Reduce Seattle Protocol Biopsies: Results From UK Multicentre Study
Source: United European Gastroenterol J. 2026 Jul 29;14(7):e70270. doi: 10.1002/ueg2.70270 (PMC13420254; doi:10.1002/ueg2.70270)
Supplement: Supplementary file 1 — Supporting Information S1 [file UEG2-14-e70270-s001.docx]

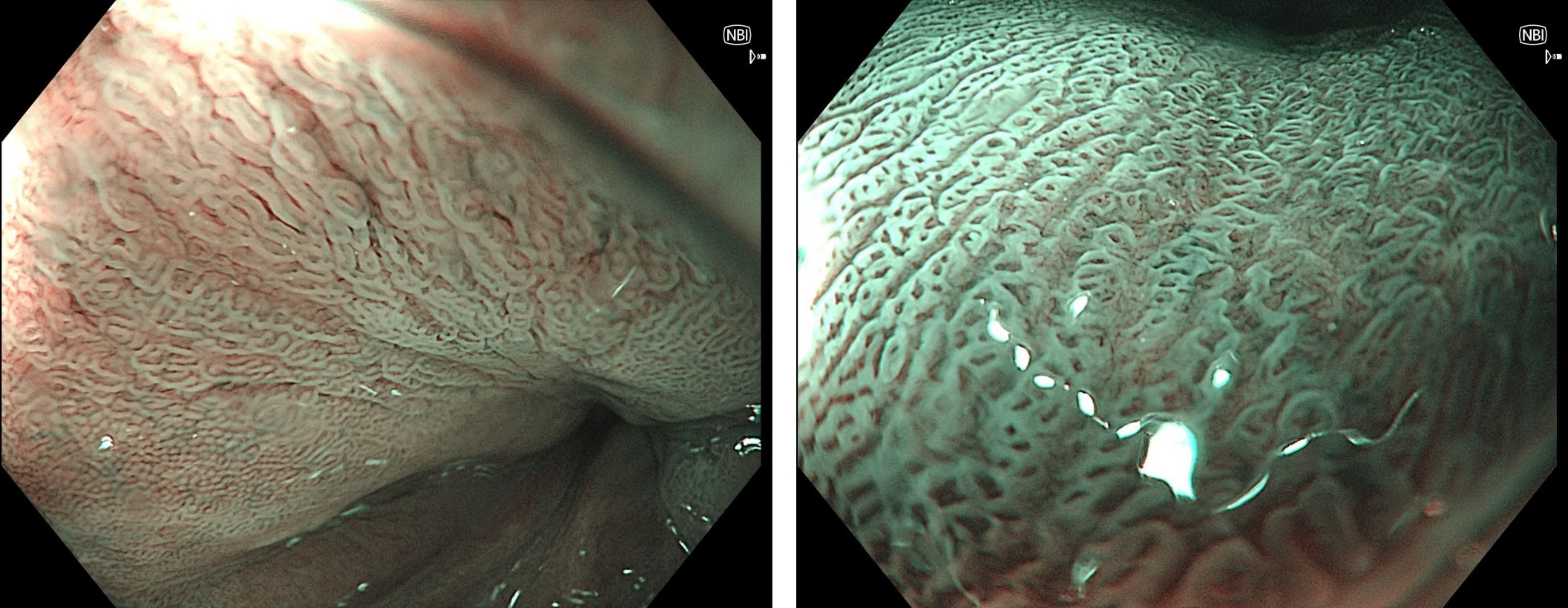


**Supplementary figure 1**. NBI in near focus (left) and NBI + AAC in near focus showing mucosal pit patterns of Barrett’s mucosa, which are regular and denotes the absence of dysplasia. Histology showed intestinal metaplasia (IM).


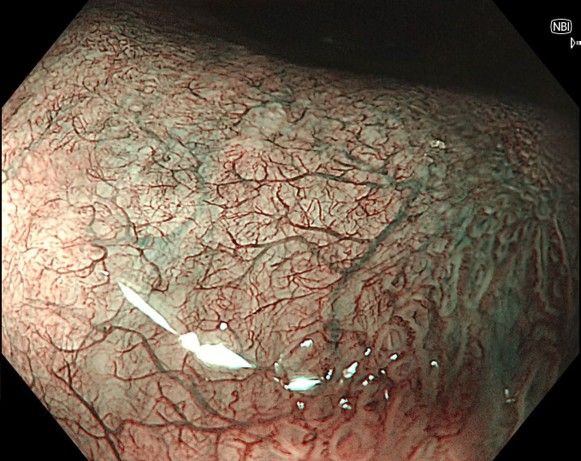


**Supplementary figure 2**. NBI with near focus showing vascular patterns of Barrett’s mucosa. Note the vessels are in normal anatomical structures without distortion to its architecture.


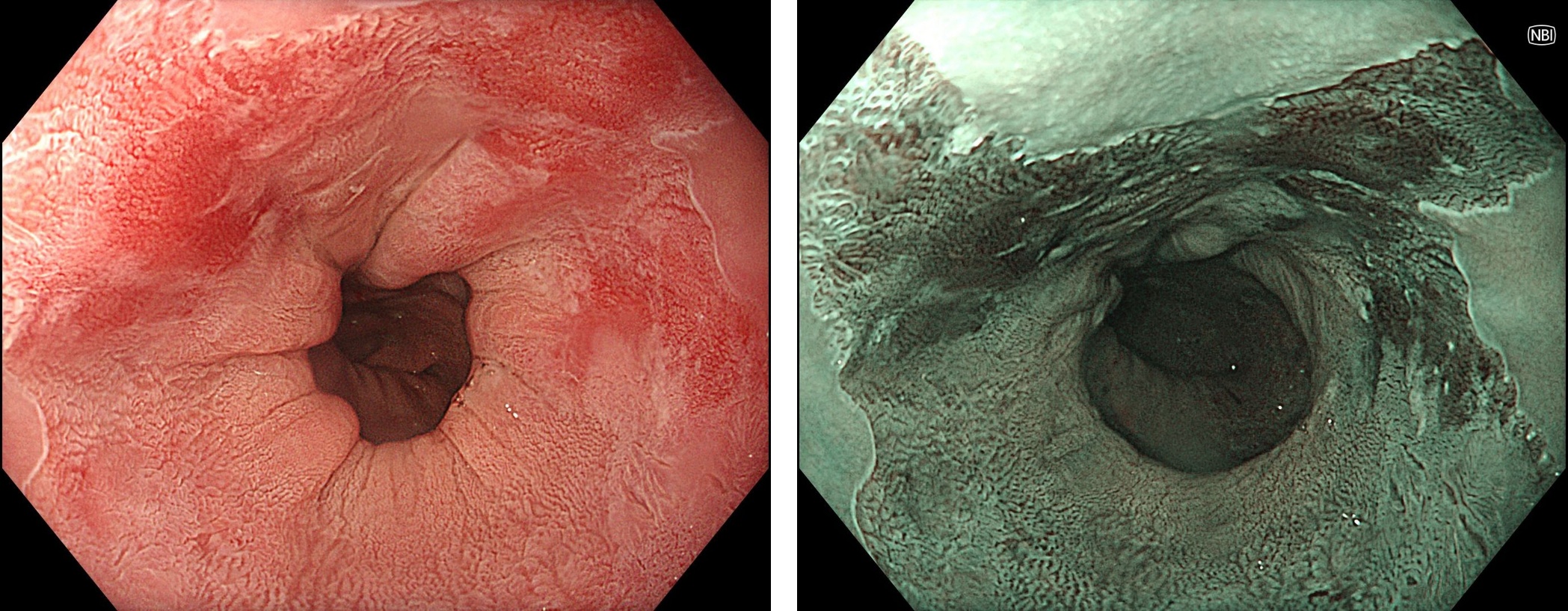


**Supplementary figure 3.** HDWLE with overwiev inspection (left) showing early loss of whittenning (ELOW) and the same in NBI + AAC mode (right). Note the ELOW patches in 1, 3 and 10 O’ clock positions which needs target biopsies. They were histologically found to be indefinite for dysplasia (IDD) and the P53 was wild type and concluded as reactive changes.


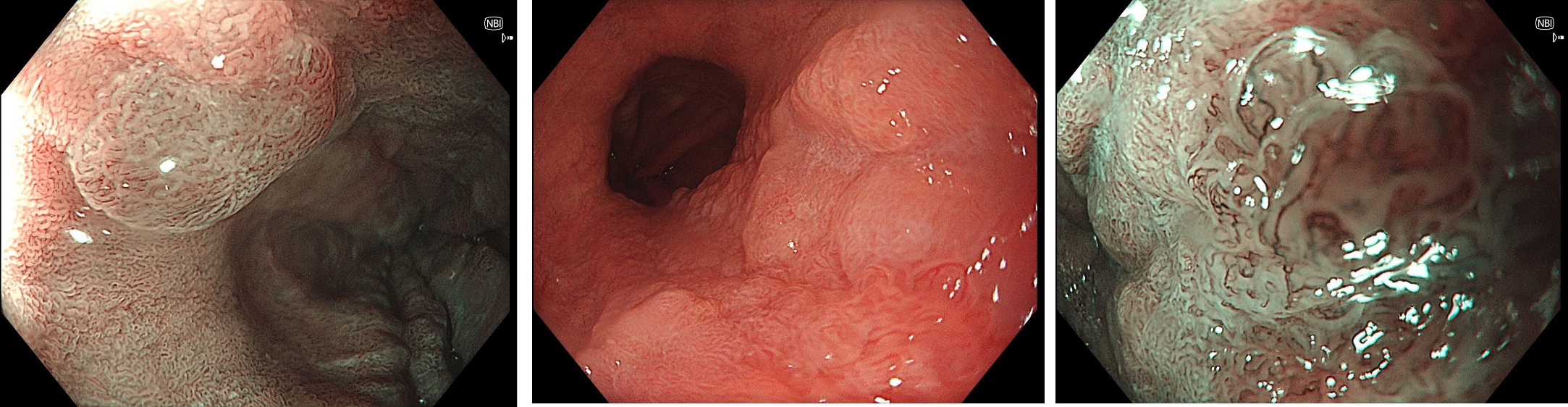


**Supplementary figure 4.** These are examples of HGD. In the left is a NBI with near focus image of BO with a polyp (Paris - Is, 5-6 mm) at 10 O’ clock position. In the middle is BO in HDWLE with overview inspection showing large irregular mucosa in 2-7 O’ clock region. Ine the left is the NBI with near view of the middle picture showing mucosal irregular pit patterns and irregular vascular patterns.
